# Programmes that Integrate Parenting Support and Financial Well-Being Support: A Systematic Scoping Review

**DOI:** 10.1007/s10935-026-00930-w

**Published:** 2026-06-29

**Authors:** Eleanor F. Bryant, Morwenna Rogers, Amy Bond, Georgia Smith, Rebecca Summers, James Hall, Kristin Liabo, Iain Lang, Vashti Berry, Nick Axford

**Affiliations:** 1 Children and Young People’s Mental Health Research Collaboration (ChYMe), University of Exeter Medical School, University of Exeter, Exeter, UK; 2 NIHR ARC South West, University of Exeter Medical School, University of Exeter, South Cloisters, St Luke’s Campus, Exeter, EX1 2LU UK; 3 Southampton Education School, University of Southampton, Southampton, UK; 4 Peninsula Medical School, NIHR ARC South West, University of Plymouth, Plymouth, UK

**Keywords:** Economic disadvantage, Financial, Parenting, Poverty, Programme, Review

## Abstract

Childhood experiences of economic disadvantage have harmful and long-lasting effects on child health. Early intervention can reduce some harms but it is unclear whether addressing economic disadvantage alongside psychosocial support yields additional benefits. This systematic scoping review aimed to identify evaluated programmes that integrate parenting support (PS) and financial well-being support (FWbS) for parents of children aged 0–19. The objectives were to describe programmes, relevant components, evaluation designs and impact on outcomes, and to consider if any programmes merit replication and testing in a UK context. Outcome or implementation evaluations of integrated PS and FWbS programmes published in English were included (no publication date or geographical limitations). A database search was supplemented by a programme registry search and consultation with parenting experts. Papers were screened independently by two reviewers. Data were extracted for: publication and study characteristics; programme description; evaluation method; measures used; and impact on outcomes (narrative description by study). Each data extraction record was checked by a second reviewer. Searches yielded 2988 (databases) and 346 (registries) articles and 20 programmes (expert consultation). After full text screening, 11 articles covering 11 programmes were included. All were targeted rather than universal. The majority principally offered PS with minimal FWbS (mostly money management). Theoretical justifications for integration of PS and FWbS were limited. Nine studies measured financial, parent health/well-being, parenting or child health/development outcomes; only two measured all four. Four studies measured implementation outcomes. Within-study impact on outcomes was mixed. Integrated PS*FWbS programmes are scarce and heterogeneous in design, content and evaluation. None should be replicated in the UK but components could be adopted and adapted in work to develop and evaluate theory-informed integrated PS*FWbS programmes.

**Supplementary Information:**

The online version contains supplementary material available at https://doi.org/10.1007/s10935-026-00930-w.

## Introduction

High levels of poverty exist worldwide. Globally, 44% of people (3.5 billion) live on less than $6.85 per day, the poverty line relevant for upper middle-income countries, while 8.5% of people (almost 700 million) are in extreme poverty (less that $2.15 per day; World Bank, 2024). Children are disproportionately affected, comprising more than 50% of those in extreme poverty despite making up just 31% of the population (Salmeron-Gomez et al., 2023). In the UK, 14.3 million people (21%) were in poverty in 2022/23, which equates to two in 10 adults and three in 10 children (Joseph Rowntree Foundation, 2025). Of course, economic disadvantage extends beyond poverty, covering a general lack of financial resources and opportunities and associated financial stressors. In the UK in 2023, for example, 54% of adults reported feeling increased anxiety or stress due to the cost of living, and 24% were found to have low financial resilience (the capacity to withstand a financial shock; Financial Conduct Authority, 2023).

Multiple studies have shown that economic disadvantage can adversely affect individuals in many ways, from the in-utero period and over the lifespan. Children from economically disadvantaged backgrounds have poorer physical and mental health, do worse in school, report lower satisfaction with their lives, are at increased risk of maltreatment and are more likely to become looked after or placed on the child protection register (Bywaters et al., 2018; Lai et al., 2019; Bennett et al., 2022; Clarke & Thévenon, 2022). As individuals grow older, experiences of childhood poverty are associated with a reduced likelihood of being in paid work or education, lower earnings and ultimately a reduced life expectancy (Bunting et al., 2018; Duncan et al., 2010; Clarke & Thévenon, 2022).

The mechanisms behind these patterns are complex but commonly understood in terms of two theories related to the family ecosystem. Family stress theory (FS) suggests that economic disadvantage creates parental stress, which can then disrupt inter-parental relationships, with increased conflict and possibly violence impairing parenting capacity (Conger et al., 2010). Resource investment theory (RI) attributes poor child outcomes to the lack of resources – specifically, money and time – that parents can invest in their child’s development, for example covering education, nutrition, clothing, housing and childcare (Duncan et al., 2014). Looking more broadly, children from poor families are exposed to greater environmental risk outside the family. They tend to live in poorer neighbourhoods that are more polluted and dangerous, with fewer high-quality services and less social cohesion, all of which increase the risk for multiple developmental outcomes (Minh et al., 2017). There are also biological pathways through which co-occurring risks related to economic disadvantage (e.g., food insecurity, infectious disease, psychological stress) interact to shape children’s neurocognitive development (Jensen et al., 2017). It is likely that a combination of mechanisms operate to produce poor child outcomes.

National social policies on employment, early childhood education, taxation and benefits can be effective in preventing or reducing child poverty (Wickham et al., 2016). Early intervention services may also have an important role to play in both improving parental capacity and reducing the pressure on families caused by economic disadvantage (Eisenstadt & Oppenheim, 2019). Until recently, however, social work and other family support services have tended not to address families’ financial situation directly, even though the families served are often on low incomes, instead focusing parent support (PS) on parent-child relationships, parenting skills or issues such as domestic violence or substance use (Morris et al., 2018; Axford & Berry, 2023).

Such PS can be effective, for instance in improving child behaviour or reducing maltreatment, but effects are often small or equivocal (Euser et al., 2015; Chen & Chan, 2016; Asmussen et al., 2022). Moreover, lower-income families are less likely to engage in such interventions for reasons that intersect with social disadvantage, such as access difficulties or stigma (Berry et al., 2022), resulting in selection effects for inequality in child outcomes (Hall et al., 2024). A broader critique of early intervention points to the failure to attend to parents’ poor material and social circumstances, questioning whether it is then feasible or ethical to expect them to enact new parenting strategies (Featherstone et al., 2016; Gillies et al., 2017). There is a case, then, for services for families – and specifically those offering PS – to attend to families’ financial circumstances as a means of improving child health and development. Our patient and public involvement work with parents supports this aspiration, indicating broadly positive views regarding service-level FWbS for families, including its integration with other services for parents, and informing the research reported in this article (Axford et al., 2026).

Some frontline services support families with their financial situation, for example through standalone financial well-being support (FWbS) services, such as income maximisation offered through Citizens Advice, while others involve integrating FWbS with social and health services, notably co-located benefits and debt advice in primary care (Beardon et al., 2021; Reece et al., 2022; Young & Bates, 2022). There is growing use of cash transfer programmes in which low-income families receive conditional or unconditional payments over a set period with the goal of reducing poverty and improving child health (Jaffee et al., 2025). A different but complementary approach called ‘poverty-aware’ or ‘anti-poverty’ practice seeks to help practitioners recognise the nature and implications of poverty and respond by challenging unjust judgements and structures and offering practical support (McCartan et al., 2018). Work to ‘poverty-proof’ services, meanwhile, involves ensuring that economically disadvantaged children are not treated differently or excluded from activities, for instance in schools (Mazzoli Smith & Todd, 2016). What is less clear is whether and how FWbS is integrated with PS, and with what impact.

The aim of this systematic scoping review was to identify evaluated programmes that integrate PS and FWbS. By ‘programme’, we mean a structured set of activities containing standardised components and running for a set period with clear progression from start to finish. Activities typically entail training and support that follow a curriculum (often manualised) and may be delivered individually or in a group, online or in-person, at home or in a service setting. We were particularly interested in finding a programme that we can replicate and test in the UK, or elements of programmes that we can incorporate in a new programme design that integrates PS and FWbS. The review focused on the nature of the programmes and relevant components, how programmes were evaluated, and their impact on outcomes. The research questions (RQ) were: (1) What programmes with integrated (components of) PS and FWbS exist? What do they do in general, and how are they delivered? (2) What do those programmes do in terms of FWbS and PS and how (if at all) are these components integrated? (3) What is the nature of evaluation studies of these (including what is measured and how, especially financial well-being)? (4) What do studies find regarding the impact on outcomes of programmes that integrate PS and FWbS?

## Methods

For this systematic scoping review, we followed the steps described in the Joanna Briggs Institute Manual for Evidence Synthesis (Chap. 10, Scoping Reviews) (Peters et al., 2024).

### Protocol and Registration

A protocol for this paper was pre-registered with Open Science Framework (OSF) and can be accessed at the following doi: https://doi.org/10.17605/OSF.IO/V6XTM. We used the Preferred Reporting Items for Systematic Reviews and Meta-Analyses extension for Scoping Reviews (PRISMA-ScR) (Tricco et al., 2018) to report this study (see checklist in Online Resource 1).

### Eligibility Criteria

Inclusion and exclusion criteria were defined using the Population, Intervention, Comparison, Outcome (PICO) Framework (Online Resource 2). We were interested in evaluations of programmes for parents of children and young people aged 0–19 years that integrate FWbS and PS. In this review we define FWbS as (1) the direct provision of money (e.g., cash, vouchers, loans) or free, subsidised or discounted goods or services (e.g., food, furniture, white goods, clothes, childcare; sometimes referred to as ‘concrete support’), and (2) services to help parents to access these and/or reduce unnecessary expenditure (e.g., income maximisation, grant writing/advocacy, money management, debt counselling). This definition aligns with what parents in the study patient and public involvement and engagement (PPIE) group told us. The term PS refers to support for parents and carers around ‘parenting skills’ and/or the parent-child relationship.

Evaluations needed to measure any of financial, parent health/well-being, parenting, child health/development or implementation outcomes; there were no other restrictions on study design. The programme needed to include PS and FWbS *content*, rather than just stating them as *goals*, and it needed to be integral to the design rather than incidental (i.e., addressed on an ad hoc basis). This distinction is not about the *amount* of support but rather whether it was *prescribed* in the programme. There were no restrictions on the balance of, or relationship between, PS and FWbS within a programme. Programmes delivered exclusively in the antenatal period, such as some involving home visiting, were ineligible. Sources had to be written in English and published in peer-reviewed or grey literature. There were no limitations by publication date or geography (although we recognise that the English language restriction may exclude some world regions).

### Information Sources and Selection of Sources of Evidence

The search strategy involved: (i) a database search; (ii) a search of programme registries; and (iii) a consultation of selected experienced international parenting support researchers. We then undertook a further focused database search (repeat of method (i)), using programme names identified in stages (i), (ii) or (iii).

Starting with databases, a search strategy was developed iteratively by MR. We used search terms for parenting or families, parenting support, finance (money, costs, vouchers etc.) and programmes. In a deviation from the protocol, we did not examine relevant reviews for included studies or conduct forward and backward citation searching. Instead, we replaced this by the registry search and consultation with parenting support researchers. We searched the following databases on 21 st and 22nd May 2024: APA PsycINFO(Ovid); ASSIA (ProQuest); Business Source Complete(EBSCOhost); CINAHL (via EBSCOhost); EconLit (EBSCOhost); EMBASE (via Ovid); ERIC (EBSCOhost); Global Health (via Ovid); HMIC (Via Ovid); IBSS (ProQuest); Social Policy and Practice (via Ovid); Sociological Abstracts (ProQuest); Sociology Database (ProQuest); and Web of Science Core Collection. The full database search strategies are available in Online Resource 3. At title and abstract stage, we double screened articles according to the PICO. Reviewer 1 was NA or AB, and reviewer 2 was EB or GS. Endnote v20 was used to host the title and abstracts for screening (The Endnote Team, 2013). We discussed any inconsistencies in a meeting involving all four reviewers. The same process was repeated at full text stage. In the case of disagreements, NA made the final decision.

We selected eight programme registries because they include a range of PS programmes in terms of geography and evidence rating (Online Resource 4). We searched registries between 13th and 30th August 2024. Filters that included financial well-being options (e.g., by outcome or risk factor) were used if available. Failing this, we used search functions for the following terms: “financ, income, cash, economic, budget, concrete”. We screened all papers returned using these methods, unless there was a high number of results for any given registry (> 60). In these cases, additional filters (e.g., setting target audience to ‘adult 18+’) were applied. The filters, search terms, results (number of programmes returned) and programmes (screened in as potentially relevant) are shown in Online Resource 5. Programmes were screened by EB, then the registry pages of those screened in by EB were checked by NA and GS.

Additionally, we contacted selected experienced international researchers of PS and asked them to identify programmes containing PS and FWbS elements. The intention was to identify additional programmes not already found through the database or registry searches and to find additional studies of programmes already identified. The consultation period was from 16th July to 6th August 2024. The names of the programmes suggested, if they had not come up previously and met the inclusion criteria, were included in stage (iv) of the search. Papers on programmes suggested by the consulted researchers did not have to meet the requirement for evidence of FWbS at title and abstract level, the logic being that the researchers may be aware of FWbS programme content not mentioned there. Papers were screened by NA and GS at full-text level using the criteria applied to the other papers.

Finally, we searched programme titles resulting from the above three methods to ensure that all relevant programmes and papers were identified. MR searched EMBASE, Social Policy and Practice, Global Health and PsycINFO on 20th September 2024. Articles were first title and abstract screened and then screened at full text by NA and EB. Results were de-duplicated against the original database search results.

### Data Charting Process

We created a data charting form in Microsoft Excel. We selected data fields to ensure coverage of the review questions. The form was calibrated by the pilot of the same two papers individually by NA, EB and MR. Descriptive data were extracted by LS, with the remaining information charted by NA or EB. Each record was checked by AB or MR. A meeting was held between the original data extractors and the checkers to discuss inconsistencies between initial data extraction and duplicate data extraction, with agreed updates made to the charted data prior to the creation of summary tables.

### Data Items

All variables for which data were sought are listed in Online Resource 6. Areas covered were: publication and study characteristics; programme description; evaluation method; measures used; and results (narrative description of within-study impact on outcomes). Measures focused on five outcome domains: financial/material; parent health/well-being; parenting; child health/development; and implementation. We used dropdown lists in the data extraction spreadsheet to allow for straightforward comparisons of papers at the analysis stage. This required assumptions to be made in the following areas: study design; type of evaluation; data collection methods; outcome measures (within the five domains); delivery setting; PS topic/content, FWbS topic/content; and mode of programme delivery. ‘Other’ categories were used in dropdown lists to cover unanticipated values.

#### Synthesis of Results

We condensed information from the data charting spreadsheet into four main tables in line with the review questions. Table 1 describes the study designs. Table 2 provides an overview of programme characteristics. Table 3 summarises the support offered by each programme, focusing on FWbS and PS. Table 4 reports which outcomes were measured. Each table was created by NA or EB and checked and amended (if necessary) by AB or MR. An additional table in Online Resource 7 gives a narrative description by study of impact on outcomes.

**Table 1:** Study designs

Author/Date	Country of study	Aim of study	Type of evaluation	Participants	Data collection methods	Outcomes measured
Benzies et al. (2012)	Canada	To examine the short-term effects of a two-generation preschool programme on parent-reported parenting stress, self-esteem and life skills for families with low income	Within-subjects pre-test/post-test	55 caregivers of 76 children Age: mean 30.3y (SD 6.5) Ethnicity: 40% Aboriginal, 60% Canadian SES: social assistance as main source of income 28%	Parent self-completion surveys	Financial well-being Parent health/well-being Parenting
Cluver et al. (2018)	South Africa	To assess the impact of ‘Parenting for Lifelong Health: Sinovuyo Teen’, a parenting programme for adolescents in low-income and middle-income countries, on abuse and parenting practices	Pragmatic cluster randomised controlled trial with built-in process evaluation	552 families (282 C and 270 I) Parent age: C 49.94y (SD = 14.2), I 48.79y (SD 15.2) Parent gender: female C 92.5%, I 97.0% SES: parent currently employed C 6.7%, I 5.2%; number of days hungry per 7 days mean C 2.88, I 2.82 Adolescent age: mean: C 13.85 (SD 2.51), I 13.83 (SD 2.26)	Parent and adolescent self-completion surveys	Financial well-being Parent health/well-being Parenting Child health/development Implementation
Dawe et al. (2003)	Australia	To ascertain the feasibility and short-term effectiveness of the Parents Under Pressure programme	Pre-post	9 families, 8 at follow-up. Age: primary caregiver mean 28.2y (SD 4.6); child 45.6 months (SD 15.4) Gender: children 6 male, 3 female	Interview (for demographic history) Parent self-report questionnaires	Parent health/wellbeing Parenting Child health/development
Katz et al. (2011)	US	To test the efficacy of paraprofessional home visitation (Pride in Parenting) to provide health and developmental intervention for high-risk African American mothers	Randomised controlled trial	286 mother infant dyads, C = 140, I = 146. Age: mother mean 25.2y C, 24.8y I Ethnicity: 98.6% African American SES: 60.1% below poverty level	Examination of hospital records Interviews Parent self-report questionnaires	Parenting Child health/development
Lachman et al. (2020)	Tanzania	To investigate the inter-relationship between economic strengthening programmes and parent management training in reducing child maltreatment	Parallel cluster randomised control trial	Total: adults 248, children 176, infants/toddlers 139 Age: adult mean 43.12y, teen 13.41y, child 11.18y Gender: adult 63.3% male, teen 60.5%, child 58.5% SES: adult not completed primary school 26.2%	Parent- and child-completed assessments	Financial well-being Parent health/well-being Parenting Child health/development
Mathias et al. (2022)	India	To examine whether the Parwarish parenting intervention was feasible, relevant and acceptable for parents and adolescents and community facilitators	Mixed methods: process evaluation and feasibility evaluation	Adult age: mother mean age 36.8y, father mean age 40.4y SES: mothers 56% never been to school, 20% class 5 completed, 18% class 10 completed, 1% class 12 completed. Child mean age: 13.3y to 14y (across 3 locations)	Interviews Focus groups	Implementation
Mebrahtu et al. (2019)	Zimbabwe	To determine the real-world effectiveness of a multicomponent community-based intervention on child development and HIV	Pragmatic parallel-arm cluster randomised controlled trial	Total 574 participants recruited, 514 retained Age: parent median age 32y (IQR 27–36), infants all 0–24 m (≥ 12 m 52% I, 50.9% C) Gender: 97.9% of caregivers biological mothers; infants 50.1% female SES: lower SES 37% I, 30% C	Self-report survey	Financial well-being Parent health/well-being Parenting Child health/development
Miller et al. (2014)	US	To tailor and implement a parenting programme with incarcerated women, and evaluate the implementation process and programme outcomes	Effectiveness-implementation hybrid	Total 45 mothers Demographic data collected for 38 participants: Age: 21 to 48y (M 31, SD 6.8) Ethnicity: 26% African-American, 63% Caucasian	Self-report survey Secondary data	Parenting Implementation
Paradis et al. (2013)	US	To evaluate programme effectiveness based on parent and child outcomes	Randomised controlled trial	Total: 497 families (270 I, 227 C) Age: mean maternal age 18.9 years (range 14–23), mean child age 6.34 months (range 1–26) Ethnicity: African American 68%, White 20%, Biracial or other 11%. Hispanic ethnicity 19%. SES: Mean annual income in $ thousands 10.9	Self-report survey Secondary data	Financial well-being Parent health/well-being Parenting Child health/development
Sadler et al. (2017)	US	To uncover preliminary information about the functions of diaper distribution and inform future research and policy questions	Qualitative	6 home visitors interviewed, who serve ~ 30 families within the programme. Ethnicity: Latina 62%, Black 28%, Mixed 10% Age: maternal age 15 to 25 years (M 19.6). SES: all families qualified for federal Women’s, Infants’ and Children’s (WIC) nutritional program supplements	Interviews with social workers who deliver the programme	Implementation
Wimer et al. (2021)	US	To examine the impacts of Room to Grow on proximal outcomes after one year	Randomised controlled trial	Total: 317 mothers (159 I, 159 C) Age: 44% 25y or under, 56% over 25y Ethnicity: 53% Hispanic, 36% Black, 4% White, 7% Other SES: 70% poverty, 72% any material hardship	Interviews Survey	Financial well-being Parent health/well-being Parenting

**Table 2:** Programme characteristics

Author/date	Name	Aim	Target group	Setting /Delivery mode	Frequency/duration	Provider(s)
Benzies et al. (2012)	Two-generation pre-school programme	To reduce the effects of day-to-day living in a family with low income by (a) providing parents with opportunities to develop new skills that increase self-esteem for parent and child and (b) provide a nurturing, caring, educational environment to assist children to reach their full potential	Parents with low income and their preschool children	Home Other [pre-school] In-person group In-person individual	Centre-based early childhood education (5 h/day for 4 days/week for 10 months, optional sessions for 2 months) Mandatory 6-week series of parenting and life skills classes at programme entry, optional sessions through year ≥ 4 home visits for family support	Calgary Urban Project Society, One World Child Development Centre, University of Calgary Certified facilitators for parenting support, social worker for home visits
Cluver et al. (2018)	Parenting for Lifelong Health (Sinovuyo Teen)	To reduce violence and improve parenting and family functioning in low-resource settings	Families reporting conflict with their adolescents aged 10–18 years	Community (not school/early years) [local community halls, churches, outdoors under trees] In-person group	14 weekly sessions: 10 jointly attended by caregivers and adolescents, 4 attended separately	Local NGO Clowns Without Borders South Africa (CWBSA) 19 locally recruited community members, 1 staff member from a regional NGO (Regional Psychosocial Support Initiative), 5 local social auxiliary workers (all trained by CWBSA)
Dawe et al. (2003)	Parents Under Pressure	To improve child behaviour, decrease parental stress and improve family functioning in methadone-maintained families by targeting affect regulation, mood, views of self as a parent, drug use and parenting skills	Parents attending a community methadone clinic, with a child aged 2–6 years	Home Other (clinic) In-person individual	10 units delivered over 12 sessions of ~ 1.5 h (exact number of sessions varies slightly but expected range 8–12) Additional phone contact as required	In association with Bialla Methadone Clinic, Brisbane, Queensland 2 therapists: 1 registered psychologist, 1 psychiatric nurse with experience in the drug and alcohol field who worked at the participating clinic
Katz et al. (2011)	Pride in Parenting	To improve knowledge, influence attitudes, and promote life skills that will assist low-income mothers in offering better health oversight and development for their infants	Low-income African American mothers aged ≥ 18 years with new babies who had used prenatal care inadequately or not at all	Home Other (not specified) In-person individual In-person group	Visits from the home visitor for 1 year (weekly birth through 4 months, biweekly 5–12 months). Biweekly parent-infant playgroups and parent discussion groups (5–12 months): 45-minute parent-infant playgroup, followed by a 45-minute parent group discussion Monthly contacts from a hospital-based social worker for 1 year postpartum	NIH-DC Initiative to Reduce Infant Mortality in Washington DC Paraprofessionals: lay home visitors drawn from the local community Groups led by a Masters-level early intervention specialist led the groups in conjunction with lay home visitors
Lachman et al. (2020)	Skilful Parenting and/or Agribusiness Training	To reduce child maltreatment by improving parenting behaviours and reducing family stress due to food insecurity and financial hardship	Parents aged ≥ 18 years from agricultural farming groups in rural Tanzania with a child aged 3–17 years	Community (not school/early years) In-person group	12 session parenting and family budgeting programme 3 sessions and ongoing support for agribusiness	Parenting programme: Professional trainers from Investing in Children and Societies (ICS; international non-profit organisation with local offices) Agribusiness: Trained staff from local economic enterprise initiative working with Ministry of Agriculture
Mathias et al. (2022)	Parwarish (adapted from Parenting for Lifelong Health: PLH-Teens)	To reduce harsh parenting and violence within families through new attitudes and skill-building between parents and adolescents	Parents living in disadvantaged communities with ≥ 1 adolescent (12–18 years), with their teens	Community (not school/early years) [not specified] Home In-person group In-person individual	Weekly parent and teen group meetings lasting 1.5–2 h, 14 modules Home visits for those who miss a weekly session	Emmanuel Hospital Association (EHA; a local non-profit organisation) Health and Development Programme teams Groups conducted by pairs of trained community facilitators (1 male, 1 female), supported by a coach
Mebrahtu et al. (2019)	Child Health Intervention for Developmental Outcomes (CHIDO)	To simultaneously enhance child stimulation, reduce economic insecurity and improve child retention in HIV care	Primary caregivers of HIV-exposed children aged 0–2 years who may experience developmental delay	Home Other (clinic) In-person group In-person individual	18 × 90-minute group sessions (parenting programme), ~fortnightly over ~ 12 months, each followed immediately by a savings and lending scheme, also monthly home visits (more frequent in cases of group non-attendance or other problems)	Bantwana Initiative of World Education Community health workers and nurses delivered the parenting programme Caregivers trained to run the savings and lending scheme Village health workers conducted home visits
Miller et al. (2014)	Parenting While Incarcerated (PWI; adaptation of Strengthening Families Program(SFP))	Not stated explicitly but SFP aims to develop parenting skills to help reduce youth depression and use of alcohol and drugs	Mothers in prison with children aged < 18 years (original for substance-abusing parents of school-aged children)	Other (prison) In-person group	Weekly 1-hour sessions over 12 weeks	Community agency serving families with an incarcerated parent (primarily mothers), also a local university Group leaders were interns from a local university and community partner agency staff who were certified to deliver SFP
Paradis et al. (2013)	Building Healthy Children (BHC)	To avoid child maltreatment, improve parent and child health, and enhance family functioning	Young low-income mothers who gave birth to their first child aged < 21 years with no previous involvement in the child welfare system	Home Other (not specified) In-person group In-person individual	Weekly parenting support (Parents as Teachers programme; PAT) home visits until child turns 3 years or until familial goals are reached Outreach visits at least bimonthly Mental health support where relevant includes child-parent psychotherapy (CPP) over a year, and/or inter-personal psychotherapy (IPT) (12 sessions)	University medical centre for outreach work Family centre for IPT and CPP Society for the Protection and Care of Children social workers for PAT BHC pediatric social worker
Sadler et al. (2017)	Minding the Baby (MTB) in partnership with The Diaper Bank	To help parents become more reflective about their own and their children’s feelings, needs and intentions, and to meet diaper need	First-time young parents in low-income families	Home In-person individual	Weekly and bi-weekly visits beginning in pregnancy and extending until the child’s second birthday	MTB and The Diaper Bank in New Haven, Connecticut MTB home visitors are nurses and social workers
Wimer et al. (2021)	Room to Grow	To support parents, infants and toddlers in the first 3 years of life, by enhancing parenting, strengthening the family’s material situation and building connections with relevant services	Parents of children aged 0–3 years (starting before child is born) who demonstrate financial need and commit to a 3-year programme	Community (not school/early years) [Room to Grow family centres] In-person individual Other [phone, text, email]	~ 2-hour sessions every 3 months over 3 years, starting with a prenatal session (total 13 sessions) Also phone/email contact between sessions	Room to Grow Room to Grow social workers

Some items here were completed by drawing in part on the study protocol article (Chingono et al., 2018)

**Table 3:** Support offered within programmes

Author/date	Programme name	Parenting support	Financial well-being support	Other support for parents	How parenting support and financial well-being support are integrated	Rationale for integration
Benzies et al. (2012)	Two-generation pre-school programme	Parent-partner relationship Other: *positive parenting behaviours*,* strategies to promote optimal child development*	Money management: *budgeting* Other: *how to apply for public transit subsidies*,* advocacy to access food and stable housing*	Life skills: managing stress, substance abuse, household and family routines, personal health and well-being, healthy pregnancy, self-esteem, job skills, cooking nutritious food on a small budget Other family support, including goal setting, counselling, accompaniment, advocacy regarding legal and child welfare systems	**Distinct topics** Budgeting (among other life skills) included within a mandatory 6-week series of parent education classes at programme entry Support with applying for subsidies and advocacy around food and housing included as part of ongoing support for parents (home visits, telephone contact)	Financial hardship adversely affects parents’ well-being (e.g., stress, frustration), which in turn affects relationships in the family, including parenting (e.g., more likely to have negative perception of child and use harsh discipline). This has negative effects on child developmental outcomes. Economic and social supports will reduce these negative effects of economic pressures.
Cluver et al. (2018)	Parenting for Lifelong Health (Sinovuyo Teen)	Parent-child relationship Family functioning Managing conflict	Money management: *budgeting*,* saving*	Managing stress and anger, protection of adolescents from community violence	**Distinct sessions** 2 of the 14 sessions cover ‘Motivation to save and making a budget with our money’ and ‘Ways to save money and making a family saving plan’ respectively	Poverty and other stressors facing families (e.g., illness, civil violence) increase disengagement and conflict, adversely affecting children. Sessions on (a) protecting adolescents from violence and exploitation in the community and (b) family financial management were added to an existing parenting programme (based on social learning theory) following participant input.
Dawe et al. (2003)	Parents Under Pressure	Parent-child relationship Parent-partner relationship Safety and basic care Managing behaviour	Money management: *budgeting*	Assessing and creating a treatment plan, challenging notion of an ideal parent, coping under pressure, coping with lapse and relapse to substance use, social support networks, life skills (inc. nutrition, health care, obtaining housing), relationships	**Distinct unit** 1 of the 10 units covers life skills (inc. budgeting)	High-risk situations that lead to drug use are linked to stress and pressure of being an isolated parent. Parenting interventions for substance-misusing families must therefore address multiple ecological domains, inc. parent/child functioning, and broader social environment (e.g., linking families with resources such as childcare and adequate housing, allowing children to attend birthday parties) to decrease life stress.
Katz et al. (2011)	Pride in Parenting	Parent-child relationship Family functioning Safety and basic care Managing behaviour	Money management: *budgeting*	Inc. postpartum women’s health needs, family planning, immunisations, reproductive health, reducing health risks, coping with stress, problem solving strategies, developing social support, involvement of fathers, healthy relationships, planning for the future, home safety, family routines, drug/alcohol use and smoking	**Distinct topics** 1 of 22 topics in the home visiting curriculum is ‘budgeting’ 1 of 10 topics for parent group discussions is ‘managing finances’	Stress of poverty interferes with parent capacity to provide a nurturing home environment for children. Also, low-income minority populations’ poor experience of health and social service systems decreases service uptake. Promoting better parenting and life skills will help low-income mothers offer better care for children.
Lachman et al. (2020)	Skilful Parenting and/or Agribusiness Training	Family functioning Safety and basic care Managing behaviour	Money management: *saving*,* communication about money* Debt reduction Support to boost income: *Access to seeds*,* advice to improve farming techniques and market connections*	Child protection, agribusiness training	**Distinct sessions/workshops** The 12-session programme includes 5 sessions on family budgeting. The agribusiness training involves 3 intensive workshops plus ongoing support	Poverty is a major risk factor for physical and emotional child abuse. Improving skills in family budgeting and saving, and increasing productivity (agricultural business), will increase family income, which in turn will improve parent well-being and reduce parent stress/depression, thereby improving family relations and decreasing child maltreatment (alongside the contribution of increasing positive parenting and non-violent age-appropriate discipline).
Mathias et al. (2022)	Parwarish [adapted version of Parenting for Lifelong Health]	Parent-child relationship Family functioning Safety and basic care Managing conflict	Money management: *budgeting*,* saving*	Keeping safe in the community, getting help and support beyond the household	**Distinct modules** The 14-module programme includes 2 modules on ‘The value of saving money and how to make a budget’	Not specified [directs reader to other studies of the programme, including Cluver et al. (2018) – see above in this table].
Mebrahtu et al. (2019)	Child Health Intervention for Developmental Outcomes	Parent-child relationship Parent-partner relationship Safety and basic care Managing behaviour	Other: *savings and lending scheme*	N/A	**Linked session** Each session on health, nutrition and early childhood stimulation is followed immediately by a savings and lending scheme session	Household economic strengthening via microfinance (e.g., loans, savings, insurance) will improve food security (and therefore child nutritional status and health) and reduce economic barriers to clinic attendance. Works alongside other family and community support to promote resilience and temper negative aspects of HIV.
Miller et al. (2014)	Parenting While Incarcerated (adaptation of Strengthening Families Program)	Parent-child relationship Safety and basic care Managing behaviour	Money management: *budgeting*	Communication, coping with addiction, custody arrangements, managing one’s own stress and anger, depression and self-esteem, grief and loss, families and alcohol, tobacco and drugs	**Distinct lesson(s)/materials** Lessons/handouts on budgeting (and other topics) developed in response to participant feedback and added to existing programme, although not clear where it sits in final curriculum	Incarcerated mothers have higher rates of unemployment and poverty. Parenting on release from prison is challenging (meeting child’s needs, managing behaviour). Prison offers a teachable moment around parenting. New content to assist with this generated at mothers’ request (inc. coping with addiction, managing stress/anger, budgeting, financial stress).
Paradis et al. (2013)	Building Healthy Children	Parent-child relationship Other: *parenting education (no detail)*	Provision of goods: *furniture*,* food*,* clothing*,* children’s items* Other: *assistance with stable and safe housing; help to secure income*,* health insurance and quality childcare*	Support with maternal educational and vocational goals; help to build social support and healthy relationships; treatment for depression; safety promotion; crisis management; assistance to negotiate healthcare and social service systems	**Parallel outreach support** Outreach workers meet with families at least bimonthly to assist with goals related to individualised service plan (inc. provision of goods and help with housing etc.); this happens alongside Parents as Teachers and/or Child Parent Psychotherapy programmes	Poverty is a risk factor for poor child health. Factors such as poor mental health, domestic violence and relationship challenges increase the risk of child maltreatment. Addressing multiple complex needs of young at-risk families will stabilise them and support compliance with medical appointments and recommended care.
Sadler et al. (2017)	Minding the Baby	Parent-child relationship Safety and basic care	Provision of goods: *diapers*	N/A	**Part of every visit** Programme home visitors routinely distribute diapers to families (according to parent need/want) in partnership with local Diaper Bank	Financial hardship affects children *directly* (health problems from diaper need, e.g., poor hydration, skin conditions) and *indirectly* (financial stress from diaper cost affects family functioning e.g., maternal guilt, poor parent-infant interactions, inability of parent to work if child cannot be in childcare). Home visiting is well placed to address multiple stressors, and a focus on diapers additionally supports parent engagement.
Wimer et al. (2021)	Room to Grow	Parent-child relationship Family functioning Safety and basic care Managing behaviour	Provision of goods: *books*,* toys*,* clothing*,* baby equipment (e.g.*,* monitors*,* bassinets*,* playards*,* strollers*,* bathtubs*,* bottles*,* breastfeeding supplies*,* cups*,* dishes*,* utensils)*	Community referrals and connections (e.g., mental health services, dental assistance, legal assistance), family planning, goal setting	**Part of every visit** Tailored 2-hour visits. First half covers topics like child development, health and safety, parent-child relationship and family system stability. Second half is the ‘baby boutique’ to jointly select new or nearly new items matching child development stage.	Children in low-income households fare worse on a wide spectrum of outcomes. Poverty adversely affects families’ ability to support children’s development by increasing stress and reducing parent well-being and human capital. Concrete assistance will enhance the effectiveness of parenting support (which aims to help parents meet children’s needs and goals for child development) by freeing up scarce resources (less focus on day-to-day needs, more on children’s long-term futures) and facilitating materials to engage in stimulating interactions. Equally, concrete assistance is more effective if coupled with parenting education.

**Table 4:** Outcomes measured

Author/date	Financial	Parent health/well-being	Parenting	Child health/development	Implementation
Benzies et al. (2012)	Other (daily life management skills, including budgeting)	Other (self-esteem)	Stress (parenting related)	None	None
Cluver et al. (2018)	Debt Financial control Other (hardship, savings, financial self-efficacy)	Mental health Alcohol/drug use	Stress (parenting related) Relationship with child Safety and basic care Other (attitudes: parent endorsement of corporal punishment)	Mental health Behavioural Other (alcohol/drug use)	Fidelity (attendance, engagement)
Dawe et al. (2003)	None	Alcohol/drug use Other (HIV risk-taking behaviour)	Stress (parenting related) Relationship with child Safety and basic care	Psychological/emotional development Behavioural	None
Katz et al. (2011)	None	None	Knowledge Stress (parenting related) Safety and basic care	Psychological/emotional development Intellectual/cognitive development	None
Lachman et al. (2020)	Other (basic child necessities, household hunger, parent attitude to budgeting)	Mental health Alcohol/drug use Relationship with partner	Skills Stress (parenting related) Relationship with child Safety and basic care Other (attitudes: parent endorsement of corporal punishment)	Physical health Psychological/emotional development Mental health Intellectual/cognitive development Behavioural Other (alcohol/drug use)	None [reported in another paper]
Mathias et al. (2022)	None	None	None	None	Acceptability Fidelity (attendance) Feasibility Other (relevance; facilitator training and coaching)
Mebrahtu et al. (2019)	Other (food security)	Physical health Mental health	Stress (parenting-related)	Physical health Physical development Intellectual/cognitive development	None
Miller et al. (2014)	None	None	Other (parenting attitudes associated with maltreatment)	None	Acceptability Appropriateness Fidelity (attendance)
Paradis et al. (2013)	Employment (parent) Other (parent education)	Mental health	Relationship with child Safety and basic care	Psychological/emotional development Social Other (child development [no detail])	None
Sadler et al. (2017)	None	None	None	None	Acceptability Fidelity (engagement)
Wimer et al. (2021)	Financial stress Other (worry about baby expenses; toys, games and books in the home)	Mental health	Skills Stress (parenting related) Safety and basic care Other (sense of competence; chaos in the home; cognitively stimulating activities)	None	None

Data are not presented in the article for all of the outcomes measured

## Results

### Selection of Sources of Evidence

**Fig. 1:**
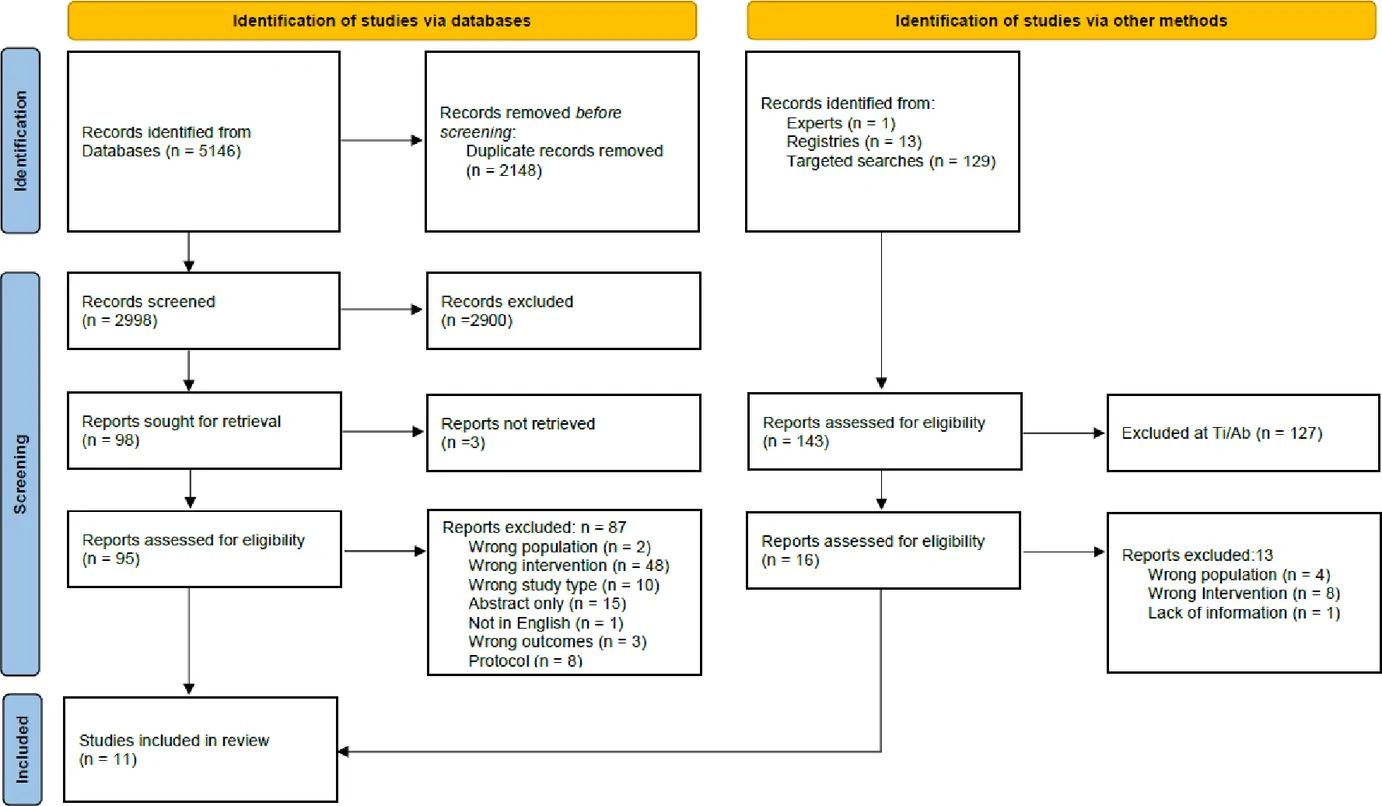
PRISMA flow diagram

Figure 1 shows the PRISMA flow diagram for the review. A total of 5146 articles were identified through database searches, with 2148 duplicates. We screened 2998 at title and abstract stage, leading to 2900 being excluded. 98 articles were sought for retrieval, and 95 were screened at the full-text stage, with 87 excluded for the following reasons: wrong intervention (*n* = 48), abstract only (*n* = 15), wrong study type (*n* = 10), protocol (*n* = 8), wrong outcomes (*n* = 3), wrong population (*n* = 2), not in English (*n* = 1). This resulted in eight included articles. Registry searches returned 346 articles. Thirteen were screened by EB for checking. After checking by NA and GS, one article was included. Sixteen parenting experts responded to our consultation, representing seven countries: UK (*n* = 7), Netherlands (*n* = 2), Sweden (*n* = 2), US (*n* = 2), Ireland (*n* = 1), Portugal (*n* = 1) and South Africa (*n* = 1). Together, they suggested 20 programmes. GS reviewed these and after exclusion of duplicates and title and abstract screening no articles were included. The programme title database search returned 129 results. At title and abstract screening, 116 papers were excluded, leaving 13 for full-text screening, two of which were included for data extraction. In total, 11 articles were included in the review.

### Characteristics of Sources of Evidence

The 11 articles identified involved 11 programmes containing both PS and FWbS elements: *Two Generation Pre-school Programme* (Benzies et al., 2012); *Parenting for Lifelong Health - Sinovuyo Teen* (Cluver et al., 2018); *Parents Under Pressure* (Dawe et al., 2003); *Pride in Parenting* (Katz, 2011), *Skilful Parenting and/or Agribusiness Training* (Lachman et al., 2020); *Parenting for Lifelong Health – Parwarish* (Mathias et al., 2022); *Child Health Intervention for Developmental Outcomes (CHIDO)* (Mebrahtu et al., 2019); *Strengthening Families – Parenting While Incarcerated (PWI)* (Miller et al., 2014); *Building Healthy Children* (BHC; Paradis et al., 2013); *Minding the Baby (MTB) in Partnership with The Diaper Bank* (Sadler et al., 2017); and *Room to Grow* (Wimer et al., 2021). Studies were published between 2012 and 2022 and conducted in seven countries: US (*n* = 5), Australia (*n* = 1), Canada (*n* = 1), India (*n* = 1), South Africa (*n* = 1), Tanzania (*n* = 1) and Zimbabwe (*n* = 1).

Study methods are summarised in Table 1. Most (*n* = 9) evaluations measured efficacy/effectiveness, with two additionally measuring feasibility or implementation. Two studies measured implementation only (Mathias, Sadler). A small majority of the evaluations (*n* = 6) used a randomised controlled trial (RCT) design (Cluver, Katz, Lachman, Mebrahtu, Paradis, Wimer), two studies each used pre-post (Benzies, Dawe) and mixed methods (Mathias, Miller), and one used qualitative methods only (Sadler). Only the RCTs had a comparison group. The articles applied a range of data collection methods, including surveys, focus groups, interviews and secondary data. The number of parents taking part in evaluations ranged from 45 to 574, although two studies reported participants as families not individuals: *n* = 9 (Dawe) and *n* = 30 (Sadler).

### Nature of Programmes [RQ1]

Programme characteristics are described in Table 2. The aims of the programmes were diverse, including, for example, reducing violence or maltreatment (Cluver, Lachman, Mathias, Paradis), improving parenting skills (Cluver, Dawe, Miller, Wimer), enhancing child development (Benzies, Katz, Mebrahtu) and addressing economic insecurity (Sadler, Wimer). All programmes had a stated aim to improve parenting or family functioning, whether by improving skills, knowledge, attitudes or behaviours, or by changing parenting practices. In contrast, only seven studies explicitly stated that the programme aimed to improve financial well-being (Benzies, Cluver, Katz, Lachman, Mebrahtu, Sadler, Wimer).^1^

The target groups for these programmes were heterogeneous. Six programmes targeted parents based on economic disadvantage, whether described as ‘low income’ (Benzies, Katz, Paradis, Sadler), in financial need (Wimer) or living in a disadvantaged community (Mathias). Each of these had additional conditions relating to one or more of child or parent age, ethnicity, parent status or service use intentions. Examples include having preschool/adolescent children, being an African American mother aged ≤ 18 years with a new baby who had used prenatal care to a limited extent or not at all, being a first-time parent, being a mother who gave birth to her first child aged under 21 years, or committing to a three-year programme. Five programmes targeted specific sub-groups: families reporting conflict with their adolescents (Cluver); parents attending a methadone clinic (Dawe); primary caregivers of HIV-exposed children (Mebrahtu); parents from agricultural farming groups in rural areas (Lachman); and mothers in prison (Miller). Collectively, the 11 programmes covered a wide child age range (0–18 years), although most had a narrower focus (e.g., 0–2 years (Mebrahtu), 10–18 years (Cluver)). Two programmes included content for parents *and* children (Cluver, Mathias) and two included a play group or home visiting session for younger children (Benzies, Katz).

All programmes were delivered in-person, either individually or in a group (or both). Two involved additional phone, email or text contact (Dawe, Wimer). In terms of setting, seven programmes took place in the family home (Benzies, Dawe, Katz, Mathias, Mebrahtu, Paradis, Sadler), four in the community (Cluver, Lachman, Mathias, Wimer), two in a clinic (Dawe, Mebrahtu), one each in a pre-school (Benzies) and prison (Miller), and two in an unspecified location (Katz, Paradis). Of the community locations, one involved halls, churches or outdoors (Cluver), one involved family centres (Wimer) and two were unspecified (Lachman, Mathias). In the six programmes delivered in multiple (two or more) settings (Benzies, Dawe, Katz, Mathias, Mebrahtu, Paradis), the family home featured in all.

Programmes generally included a set number of sessions (range 3 to > 50), although the frequency of delivery varied. A small majority of programmes (*n* = 6) involved 10–18 sessions delivered weekly, biweekly or monthly (Cluver, Dawe, Lachman, Mathias, Mebrahtu, Miller). The two longest programmes contained weekly and biweekly visits from pregnancy until the child’s 2nd birthday (Sadler) or weekly visits from as young as one month until the child’s 3rd birthday (Paradis).

### Nature of FWbS, PS and Integration [RQ2]

Table 3 outlines the nature of support and the type of integration of FWbS and PS. In some instances other support was also provided, for instance on family planning, immunisations, drug/alcohol use and managing stress.

All six types of PS content pre-specified in our data extraction framework were offered across the programmes. The most common were improving the parent-child relationship (*n* = 9; Cluver, Dawe, Katz, Mathias, Mebrahtu, Miller, Paradis, Sadler, Wimer) and safety and basic care (*n* = 8; Dawe, Katz, Lachman, Mathias, Mebrahtu, Miller, Sadler, Wimer). These were followed by managing behaviour (*n* = 6; Dawe, Katz, Lachman, Mebrahtu, Miller, Wimer) and family functioning (*n* = 5; Cluver, Katz, Lachman, Mathias, Wimer). Three programmes included content on the parent-partner relationship ( Benzies, Dawe, Mebrahtu), while two offered advice on managing conflict (Cluver, Mathias). Two programmes offered support that did not fit pre-specified categories, namely positive parenting behaviours and strategies to promote optimal child development (Benzies) and parenting education (Paradis). All programmes provided PS on two or more (up to four) topics.

FWbS largely entailed money management (budgeting, saving, communication about finance), with seven programmes including this (Benzies, Cluver, Dawe, Katz, Lachman, Mathias, Miller). Three programmes included the provision of goods (Paradis, Sadler, Wimer), while debt reduction (Lachman), support to boost income (Lachman) and a savings and lending scheme (Mebrahtu) appeared once each. Only three of the 11 programmes offered more than one kind of FWbS: in Benzies, parents were offered support to apply for public transport subsidies and advocacy to access food and stable housing; in Lachman, money management advice and debt reduction were included in the parenting arm of the trial and support to boost income additionally included in the agribusiness and parenting arm; and in Paradis, an outreach worker offered assistance with housing and help to secure income, health insurance and quality childcare. None of the programmes included provision of cash in the form of topping up a family’s income.

The most common method of integrating FWbS within a programme was by having distinct modules, units, sessions, topics, lessons or materials on the subject (*n* = 7; Benzies, Cluver, Dawe, Katz, Lachman, Mathias, Miller). In two programmes, FWbS was part of every home visit, either according to parent need and want (Sadler) or as the second half of visit in which the first half focused on PS (Wimer). In one programme, outreach workers provided the FWbS in home visits that ran parallel to the PS home visits (Paradis). The final programme (Mebrahtu) was a hybrid, with FWbS provided as a linked session immediately after each PS session.

Most studies acknowledged the contribution of financial hardship to child outcomes, with some (mostly limited) articulation of the mechanisms through which this occurs. These mostly map onto FS theory, for instance the impact on family conflict, family stress, family functioning, parent well-being (especially mental health), parenting and parent-child interactions (Benzies, Cluver, Sadler, Wimer). Fewer map onto RI theory, for example a lack of goods directly affecting child physical health (Wimer) and poverty-related stress interfering with parents’ capacity to provide a nurturing home environment (Katz).

Most studies also acknowledged that attending to families’ social context, including financial and material issues, will help children. However, mechanisms for this were spelled out more in some cases than in others. Among the fuller accounts, Lachman et al. (2020) described boosting financial well-being to reduce parent stress, thereby improving family relations and reducing child maltreatment (FS theory), while Mebrahtu et al. (2019) explained how better financial skills and increased productivity will improve food security and therefore children’s health and nutritional status (RI theory). Similarly, Wimer et al. (2021) outlined how concrete assistance will facilitate materials for engaging in stimulating parent-child interactions (RI theory) while also freeing up parental capacity to focus less on day-to-day needs and more on the long term (FS theory). Another mechanism cited is FWbS helping reduce barriers to service use (Mebrahtu). Only two studies detailed how mechanisms triggered by FWbS dovetail with those triggered by PS. Lachman et al. (2020) presented a theory of change showing how improved positive parenting (from PS) and increased parent well-being (from FWbS) both contribute to better family relations and therefore reduced child maltreatment. Meanwhile, Wimer et al. (2021) advised that “parent coaching and referrals will be more effective if families are also receiving substantial material goods and similarly that the material goods will be more beneficial if they are coupled with parenting education and community connections” (p.3). In two cases (Cluver, Miller) the inclusion of FWbS in a PS programme was explained as resulting directly from participant input.

### Nature of Programme Evaluations and Outcomes Measured [RQ3]

Almost all evaluations (*n* = 10) measured outcomes through parent and/or child self-report, whether through questionnaires, interviews or focus groups, with three of these additionally using secondary data (Katz, Miller, Paradis); the one study (Sadler) that did not collect data from parents or children obtained the views of practitioners delivering the programme. The outcomes measured in the studies are shown in Table 1 (domains) and Table 4 (detail). Nine studies measured and reported on financial well-being, parent health/well-being, parenting and/or child health/development outcomes (Benzies, Cluver, Dawe, Katz, Lachman, Mebrahtu, Miller, Paradis, Wimer). Of these, six measured financial well-being (Benzies, Cluver, Lachman, Mebrahtu, Paradis, Wimer), seven measured parent health/well-being (Benzies, Cluver, Dawe, Lachman, Mebrahtu, Paradis, Wimer), nine measured parenting (Benzies, Cluver, Dawe, Katz, Lachman, Mebrahtu, Miller, Paradis, Wimer) and six measured child health/development outcomes (Cluver, Dawe, Katz, Lachman, Mebrahtu, Paradis). Two studies reported implementation outcomes only (Mathias, Sadler), with two reporting implementation alongside other outcomes (Cluver, Miller).

The six studies that examined financial well-being outcomes all did so differently. Benzies et al. (2012) measured daily life management skills, including budgeting. Cluver et al. (2018) examined financial control, debt, hardship, savings and financial self-efficacy. Lachman at al. (2020) measured household hunger, basic child necessities (e.g., clothing, school supplies) and parent attitude to budgeting. Mebrahtu et al. (2019) looked at household hunger and food deprivation. Paradis et al. (2013) measured parent employment. Wimer et al. (2021) measured financial stress, worry about baby expenses and the presence of toys, books and games in the home. No study measured income or material living standards.

Out of the seven studies that measured parent health/well-being outcomes, six measured mental health (Benzies, Cluver, Lachman, Mebrahtu, Paradis, Wimer) and three measured alcohol or drug use (Cluver, Dawe, Lachman). Parent physical health (Mebrahtu), parent relationship with partner (Dawe) and parent self-esteem (Benzies) were each measured once.

Parenting outcomes were the most measured outcome, occurring in nine studies. Parenting-related stress was the most common (*n* = 7; Benzies, Cluver, Dawe, Katz, Lachman, Mebrahtu, Sadler), followed by safety and basic care (*n* = 6; Cluver, Dawe, Katz, Lachman, Paradis, Wimer), which in some cases included reports of abusive or harsh parenting. Parent-child relationship was measured in four studies (Cluver, Dawe, Lachman, Paradis). Two studies measured parenting skills (Lachman, Wimer) and one measured parenting knowledge (Katz). Other parenting outcomes measured were parenting attitudes (Cluver, Lachman, Miller), which included endorsement of corporal punishment or attitudes associated with maltreatment, and sense of competence, chaos in the home and provision of cognitively stimulating activities (all Wimer).

Child health/development was measured in six studies, covering a range of outcomes. Four studies measured psychological/emotional development (Dawe, Katz, Lachman, Paradis), while three studies respectively measured intellectual/cognitive development (Dawe, Katz, Mebrahtu) and behaviour (Cluver, Dawe, Lachman). Two studies measured each of physical health (Lachman, Mebrahtu) and mental health (Cluver, Lachman). One study each measured physical development (Mebrahtu) and social development (Paradis). None of the studies measured educational outcomes. One child health/development outcome measure we had not anticipated, coded under ‘other’, was alcohol/drug use (Cluver, Lachman).

Four studies evaluated implementation outcomes. The outcomes that were measured varied, with the most common being fidelity (*n* = 4; Cluver, Mathias, Miller, Sadler), acceptability (*n* = 3; Mathias, Miller, Sadler), engagement (*n* = 2; Mathias, Sadler) and session attendance (*n* = 2; Cluver, Mathias). Feasibility, relevance, appropriateness and facilitator training and coaching were measured in one study (Mathias).

### Impact of Programmes on Outcomes [RQ4]

Outcome results are described narratively in Online Resource 7. All studies reported positive effects for some or all outcomes, although results within studies were often mixed between outcomes or within outcome domains. Here we report briefly on programmes offering the most substantial FWbS (all evaluated by RCT). Cluver et al. (2018) was arguably the most promising, with positive effects for economic hardship, caregiver mental health, some aspects of parenting (physical and emotional abuse but not neglect or inconsistent discipline) and child substance use but not externalising behaviour. In Lachman et al. (2020), there were no effects on household hunger or family budgeting, no effects on parent mental health, positive effects on some aspects of parenting (including maltreatment), and mixed effects on child outcomes (e.g., reduced behaviour problems, no effects on early childhood development). In an analysis of proximal outcomes (12 months into a three-year programme), Wimer et al. (2021) found a positive effect on toys, goods and books in the home, no effect on parent financial worries or parent mental health, and positive effects on some parenting outcomes (e.g., sense of competence, parenting stress).

### Summary of Findings

We identified 11 relevant programmes. These originated from both the Global North (*n* = 7) and the Global South (*n* = 4), although the nature of programmes did not obviously vary along this axis. The majority principally offered PS with minimal FWbS. The FWbS was typically a discrete component rather than woven throughout the PS content, and theoretical justifications for the within-programme integration of FWbS and PS were mostly fairly limited. Money management was the most common form of FWbS, and only three programmes offered more than one type of FWbS. We did not identify any programme that gave families money. No programme was universal; seven targeted families based on some metric of economic disadvantage, and four focused on specific population groups. In terms of evaluation, nine studies (including six RCTs) measured financial, parent, parenting or child outcomes, the most popular outcome area being parenting (*n* = 9). The impact of the programmes on financial well-being was only measured in six studies, and then inconsistently; none of these used the same constructs, let alone measures. Only three studies measured impact in all four of the financial well-being, parent health/well-being, parenting and child health/development outcome areas, limiting the ability to trace mechanisms of impact via the FS or RI theories. Outcomes were generally measured over a short period (typically pre-post intervention), making it difficult to determine longer-term impact. The impact of each programme on outcomes, as reported by study authors, was often mixed within and across outcome domains.

## Discussion

This systematic scoping review aimed to identify evaluated programmes integrating PS and FWbS, describe them and their respective PS and FWbS components, outline how the programmes were evaluated and describe individual programme impact on outcomes. Our assessment is that currently there is no established evidence-based programme that integrates PS and FWbS in a way that merits replication in the UK. The FWbS offered in most programmes was relatively slight. Two of the three programmes with more substantial FWbS (Cluver, Lachman) have so far only been delivered and tested in low- and middle-income countries (LMICs). Trial evidence for the other, the three-year Room to Grow programme (Wimer), which is being tested in the US and includes up to $10,000 material support for the baby, is so far restricted to proximal 12-month outcomes. Reported outcomes within these studies were mixed. However, elements of these and other programmes could be adopted and adapted as part of an intervention design/optimisation process. In doing so there are several considerations, all of which need exploring with parents and practitioners.

The first is the extent to which such a programme is targeted. All programmes in this review were targeted, in many cases based on indicators of economic disadvantage, but there is scope to integrate FWbS into universal PS. The advantages of this include enabling easier access to support and helping to avert serious financial problems. Applying the principle of proportionate universalism (Marmot, 2010; Marmot et al., 2020) would mean that support is available to all but some families receive extra help to meet additional (sometimes acute) needs.

Second, it will be vital to consider the nature and duration of FWbS offered and how this relates to families’ needs and context. For instance, some families may need intensive short-term crisis support in the form of cash or goods, whereas others may benefit from longer-term help with money management. Similarly, assistance with income maximisation may not benefit a family that already claims the benefits to which they are entitled.

Third is the question of how much FWbS can reasonably be integrated into a programme that focuses primarily on PS. In most programmes considered here it was minimal, despite being core to the intervention design. Offering more FWbS might be beneficial, indeed there are theoretical arguments for this (see below) and integrating two interventions in one can potentially increase the efficiency of delivery and minimise implementation fatigue (Domitrovich et al., 2010). Equally, it may make a programme unwieldy to deliver and, in the case of fusing PS and FWbS, encounter resistance from both parents (stigma) and practitioners (lack of financial expertise, reluctance to stray beyond traditional professional remit). Navigating these complexities in intervention design will require strong engagement with practitioners and parents.

Fourth, careful thought needs to be given to both the logistics and logic of integrating PS and FWbS. On logistics, one approach is to add modules, sessions or elements, the most common model in the studies in this review. An alternative is to weave additional content throughout, so that PS and FWbS are intertwined. These two methods of integration have been described as ‘bundling’ and ‘blending’ respectively (Villalobos & Chambers, 2023). On programme logic, there is potential to achieve additive and synergistic effects from fusing independent strategies or ingredients to achieve common outcomes (Domitrovich et al., 2010). Some studies in this review indicate the possibility of integrating mechanisms triggered by PS and FWbS respectively (including FS and RI theories) in a single framework, although there is scope to go deeper.

A fifth consideration for programme design and optimisation is the delivery mode. All programmes we identified were delivered in person, so there is scope to explore the potential for online delivery of at least some content. A recent meta-analysis found that when professional guidance is present, online PS is an effective, non-inferior alternative to in-person support and may actually be preferred by parents (Leijten et al., 2024). It concluded that further research is needed, however, for example to understand the conditions under which parents prefer and potentially benefit more from online support over in-person support, and the conditions that online support must meet in order to be non-inferior to in-person support. The acceptability and effectiveness of offering online FWbS in the context of PS would need to be investigated. Initial indications, based on feedback from our PPIE group, are that a mix of in-person and digital support would make services more accessible to a range of different parents – for example, those who work during the day.

The findings of our review have implications for research. Studies need to provide stronger descriptions of programme elements, particularly FWbS. There is also a need for RCTs with integrated implementation and process evaluations that measure programme effectiveness in terms of child outcomes but also mechanisms of impact. This requires at the very least measuring core constructs along the theorised pathways to impact on child outcomes, including financial well-being, parent health/well-being and parenting (to cover the FS/RI theories). Exploring other hypothesised mechanisms, such as the impact on families’ housing and neighbourhood, would necessarily require measuring additional constructs. Undertaking such research using a similar framework of constructs in different places will assist with understanding the extent to which mechanisms hold across contexts (e.g., Global South vs. Global North). Careful consideration of how to measure financial well-being adequately and consistently is needed, using both objective and subjective indicators. Parents in the PPIE group indicated some discomfort about providing data on financial well-being, so this needs to be handled sensitively. The precise constructs and measures will necessarily be informed by programme nature and focus. For example, a programme might be less concerned with boosting income or enhancing parenting skills and more focused on reducing financial and parenting-related stress.

This review has several strengths and limitations. One strength is that all stages of a scoping review methodology have been followed systematically using international guidelines for conduct. While it was difficult to create search terms that would capture all relevant papers, this was mitigated by PPIE group input on terminology and the researcher consultation and registry search. A limitation is that we did not include PS programmes where FWbS is offered incidentally, or those that focus on employment support. We have reported thoroughly on the nature of the programmes and their respective FWbS and PS components, notwithstanding the limited detail on these in most articles. As this is a scoping review we have reported outcome results descriptively only, with no synthesis of findings or critical appraisal of study methods.

## Conclusions

Programmes that integrate PS and FWbS are uncommon. The 11 programmes identified in this systematic scoping review were heterogeneous in design and content. They were also evaluated inconsistently, with scant attention to mechanisms of impact. Our assessment is that none should be replicated in the UK (or, indeed, other similar high-income countries). We base this on three factors: the often relatively minimal and one-dimensional nature of much FWbS offered, and associated often limited theoretical justification for its integration with PS; challenges around cultural transferability, notably for programmes developed and tested in LMIC contexts; and the lack of compelling evidence of effectiveness (owing to study design and/or mixed reported outcomes within studies). However, elements of some programmes could be adopted and adapted. Further work to develop integrated PS*FWbS programmes in a new context requires collaborating with practitioners and, critically, economically disadvantaged parents. Such parents and their families experience stigma in multiple ways, including through well-meaning policies, charitable efforts and research. Studies in this space might not intend to cause ‘othering’ but nevertheless can have this impact. Conducting research and policy making in this area therefore requires sensitivity and the involvement of parents who are directly affected. Finally, any integrated PS*FWbS programme needs to be tested in an RCT – ideally facilitating comparison between integrated support and standalone PS – with mixed methods process evaluations to determine implementability, effectiveness and mechanisms of impact.

## Electronic Supplementary Material

Below is the link to the electronic supplementary material.

Supplementary material 1 123 kb

## Footnotes

1 The others did not state plainly the aim of including FWbS. Of these, Mathias points readers to other papers for programme information, including the Cluver study in this review (which does state the aim).
