# Supplementary material for: Programmes that Integrate Parenting Support and Financial Well-Being Support: A Systematic Scoping Review
Source: J Prev (2022). 2026 Jun 29;47(6):1057–90. doi: 10.1007/s10935-026-00930-w (PMC13759693; doi:10.1007/s10935-026-00930-w)
Supplement: Supplementary file 1 [file 10935_2026_930_MOESM1_ESM.docx]

**Online Resource 1: Preferred Reporting Items for Systematic reviews and Meta-Analyses extension for Scoping Reviews (PRISMA-ScR) Checklist^[1]^**

| **SECTION** | **ITEM** | **PRISMA-ScR CHECKLIST ITEM** | **REPORTED ON PAGE #** |
| --- | --- | --- | --- |
| **TITLE** |  |  |  |
| Title | 1 | Identify the report as a scoping review. | 1 |
| **ABSTRACT** |  |  |  |
| Structured summary | 2 | Provide a structured summary that includes (as applicable): background, objectives, eligibility criteria, sources of evidence, charting methods, results, and conclusions that relate to the review questions and objectives. | 2 |
| **INTRODUCTION** |  |  |  |
| Rationale | 3 | Describe the rationale for the review in the context of what is already known. Explain why the review questions/objectives lend themselves to a scoping review approach. | 3-6 |
| Objectives | 4 | Provide an explicit statement of the questions and objectives being addressed with reference to their key elements (e.g., population or participants, concepts, and context) or other relevant key elements used to conceptualize the review questions and/or objectives. | 6 |
| **METHODS** |  |  |  |
| Protocol and registration | 5 | Indicate whether a review protocol exists; state if and where it can be accessed (e.g., a Web address); and if available, provide registration information, including the registration number. | 6 |
| Eligibility criteria | 6 | Specify characteristics of the sources of evidence used as eligibility criteria (e.g., years considered, language, and publication status), and provide a rationale. | 6-7 |
| Information sources* | 7 | Describe all information sources in the search (e.g., databases with dates of coverage and contact with authors to identify additional sources), as well as the date the most recent search was executed. | 7-9 |
| Search | 8 | Present the full electronic search strategy for at least 1 database, including any limits used, such that it could be repeated. | Online Resource 3 |
| Selection of sources of evidence† | 9 | State the process for selecting sources of evidence (i.e., screening and eligibility) included in the scoping review. | 7-9 |
| Data charting process‡ | 10 | Describe the methods of charting data from the included sources of evidence (e.g., calibrated forms or forms that have been tested by the team before their use, and whether data charting was done independently or in duplicate) and any processes for obtaining and confirming data from investigators. | 9-10 |
| Data items | 11 | List and define all variables for which data were sought and any assumptions and simplifications made. | 10 |
| Critical appraisal of individual sources of evidence§ | 12 | If done, provide a rationale for conducting a critical appraisal of included sources of evidence; describe the methods used and how this information was used in any data synthesis (if appropriate). | N/A |
| Synthesis of results | 13 | Describe the methods of handling and summarizing the data that were charted. | 10 |

| **RESULTS** |  |  |  |
| --- | --- | --- | --- |
| Selection of sources of evidence | 14 | Give numbers of sources of evidence screened, assessed for eligibility, and included in the review, with reasons for exclusions at each stage, ideally using a flow diagram. | 11 |
| Characteristics of sources of evidence | 15 | For each source of evidence, present characteristics for which data were charted and provide the citations. | 11-12 |
| Critical appraisal within sources of evidence | 16 | If done, present data on critical appraisal of included sources of evidence (see item 12). | N/A |
| Results of individual sources of evidence | 17 | For each included source of evidence, present the relevant data that were charted that relate to the review questions and objectives. | 12-17 |
| Synthesis of results | 18 | Summarize and/or present the charting results as they relate to the review questions and objectives. | 12-17 |
| **DISCUSSION** |  |  |  |
| Summary of evidence | 19 | Summarize the main results (including an overview of concepts, themes, and types of evidence available), link to the review questions and objectives, and consider the relevance to key groups. | 17-20 |
| Limitations | 20 | Discuss the limitations of the scoping review process. | 20 |
| Conclusions | 21 | Provide a general interpretation of the results with respect to the review questions and objectives, as well as potential implications and/or next steps. | 20-21 |
| **FUNDING** |  |  |  |
| Funding | 22 | Describe sources of funding for the included sources of evidence, as well as sources of funding for the scoping review. Describe the role of the funders of the scoping review. | 21 |

JBI = Joanna Briggs Institute; PRISMA-ScR = Preferred Reporting Items for Systematic reviews and Meta-Analyses extension for Scoping Reviews.

* Where *sources of evidence* (see second footnote) are compiled from, such as bibliographic databases, social media platforms, and Web sites.

1. A more inclusive/heterogeneous term used to account for the different types of evidence or data sources (e.g., quantitative and/or qualitative research, expert opinion, and policy documents) that may be eligible in a scoping review as opposed to only studies. This is not to be confused with *information sources* (see first footnote).
2. The frameworks by Arksey and O’Malley (6) and Levac and colleagues (7) and the JBI guidance (4, 5) refer to the process of data extraction in a scoping review as data charting*.*
3. The process of systematically examining research evidence to assess its validity, results, and relevance before using it to inform a decision. This term is used for items 12 and 19 instead of "risk of bias" (which is more applicable to systematic reviews of interventions) to include and acknowledge the various sources of evidence that may be used in a scoping review (e.g., quantitative and/or qualitative research, expert opinion, and policy document).

*From:* Tricco AC, Lillie E, Zarin W, O'Brien KK, Colquhoun H, Levac D, et al. PRISMA Extension for Scoping Reviews (PRISMAScR): Checklist and Explanation. Ann Intern Med. 2018;169:467–473[. doi: 10.7326/M18-0850.](http://annals.org/aim/fullarticle/2700389/prisma-extension-scoping-reviews-prisma-scr-checklist-explanation)

**Online Resource 2: Inclusion and exclusion criteria**

| **Include** | **Exclude** |
| --- | --- |
| *Population* | |
| - Families with children and young people aged 0-19 years, where parents/carers at minimum receive support (other family members may also receive support) - No restriction by geography | - Support for children / young people but not their parents/carers - Support for adults who may or may not be parents/carers (e.g., programme for low-income adults aged 25-65 years) - Support offered in the antenatal period only |
| *Intervention* | |
| - A programme with integrated parenting support and financial well-being support - The parenting and financial well-being support are core parts of the programme (e.g., included in the programme description and/or logic model) - Support may be provided as standalone sessions / content or woven in throughout - Could be any balance of parenting and financial well-being support (i.e., primarily a parenting programme with some financial support added in, or primarily a financial support programme with some parenting added in) - Universal (i.e., parents/carers irrespective of status / need) or targeted based on status / need (e.g., living in a low-income area, child has behavioural difficulties) | - An intervention that includes parenting and financial well-being support but not in the form of a programme - A parenting programme that doesn’t offer financial well-being support - A financial well-being support programme that doesn’t offer any parenting support - Financial well-being support offered primarily to enable or support attendance at the parenting programme (e.g., food, transport, childcare, financial incentives) - Financial well-being support that is incidental (i.e., not a core part of the intervention but rather offered on an ad hoc basis) |
| *Comparison* | |
| - The study can be any form of evaluation of the programme (i.e., no comparison group needed) | - A description only of the programme (i.e., not an evaluation) |
| *Outcomes* | |
| - Financial well-being (e.g., income, debt, financial stress) - Parent health / well-being (physical / mental) - Parenting (attitudes, knowledge, skill) - Child health / development (physical, mental, social, psychological, educational) - Implementation (e.g., acceptability, fidelity) | - N/A |

**Online Resource 3: Database search strategies**

EMBASE/PsycINFO/Global Health

1	((parenting or parents or parent or mother* or father* or family or families) and (support* or help* or assist* or guidance or advice or counselling)).ti.	13019

2	home visit*.ti. and (parenting or parents or parent or mother* or father* or family or families or child*).ti,ab.	1031

3	(parenting and (program* or intervention or initiative or project or model or training or class* or education)).ti.	2617

4	((financ* or money or debt or household or income or wellbeing or concrete or material or food) and (support* or help* or assist* or guidance or advice or counselling)).ti,ab.	115511

5	personal finance/	311

6	financ*.ti,ab.	71717

7	microfinanc*.ti,ab.	344

8	debt.ti,ab.	3514

9	money.ti,ab.	20044

10	income.ti,ab.	77895

11	economic.ti,ab.	118129

12	cash.ti,ab.	3440

13	budget*.ti,ab.	10291

14	voucher*.ti,ab.	1006

15	expenditure.ti,ab.	8905

16	cost.ti,ab.	76196

17	goods.ti,ab.	8633

18	or/5-17	340170

19	(program* or intervention* or initiative* or project* or scheme* or model* or training).ti,ab.	1884271

20	1 and 4 and 18 and 19	672

21	3 and 18	375

22	2 and 18 and 19	179

23	20 or 21 or 22	1195

Cinahl/ERIC/Business Source Complete/EconLit

| S24 | S21 OR S22 OR S23 |
| --- | --- |
| S23 | S4 AND S19 AND S20 |
| S22 | S5 AND S19 |
| S21 | S1 AND S6 AND S19 AND S20 |
| S20 | TI ( "program*" or "intervention*" or "initiative*" or "project*" or "scheme*" or "model*" or "training" ) OR AB ( "program*" or "intervention*" or "initiative*" or "project*" or "scheme*" or "model*" or "training" ) |
| S19 | S7 OR S8 OR S9 OR S10 OR S11 OR S12 OR S13 OR S14 OR S15 OR S16 OR S17 OR S18 |
| S18 | TI goods OR AB goods |
| S17 | TI cost OR AB cost |
| S16 | TI expenditure OR AB expenditure |
| S15 | TI voucher* OR AB voucher* |
| S14 | TI budget* OR AB budget* |
| S13 | TI cash OR AB cash |
| S12 | TI economic OR AB economic |
| S11 | TI income OR AB income |
| S10 | TI money OR AB money |
| S9 | TI debt OR AB debt |
| S8 | TI microfinanc* OR AB microfinanc* |
| S7 | TI financ* OR AB financ* |
| S6 | TI ( (("financ*" or "money" or "debt" or "household" or "income" or "wellbeing" or "concrete" or "material" or "food") and ("support*" or "help*" or "assist*" or "guidance" or "advice" or "counselling")) ) OR AB ( (("financ*" or "money" or "debt" or "household" or "income" or "wellbeing" or "concrete" or "material" or "food") and ("support*" or "help*" or "assist*" or "guidance" or "advice" or "counselling")) ) |
| S5 | TI (("parenting" and ("program*" or "intervention" or "initiative" or "project" or "model" or "training" or "class*" or "education"))) |
| S4 | S2 OR S3 |
| S3 | TI "home visit" AND AB ( "parenting" or "parents" or "parent" or "mother*" or "father*" or "family" or "families" or "child*" ) |
| S2 | TI "home visit" AND TI ( "parenting" or "parents" or "parent" or "mother*" or "father*" or "family" or "families" or "child*" ) |
| S1 | TI ((("parenting" or "parents" or "parent" or "mother*" or "father*" or "family" or "families") and ("support*" or "help*" or "assist*" or "guidance" or "advice" or "counselling"))) |

ASSIA, IBSS, Sociological Abstracts, Sociology database

((title("parenting" OR "parents" OR "parent" OR "mother*" OR "father*" OR "family" OR "families") AND title("support*" OR "help*" OR "assist*" OR "guidance" OR "advice" OR "counselling")) AND (title("financ*" OR "money" OR "debt" OR "household" OR "income" OR "wellbeing" OR "concrete" OR "material" OR "food") AND title("support*" OR "help*" OR "assist*" OR "guidance" OR "advice" OR "counselling")) AND title("financ*" OR "microfinanc*" OR "debt" OR "money" OR "income" OR "economic" OR "cash" OR "budget*" OR "voucher" OR "expenditure" OR "cost" OR "goods") AND (title("program*" OR "intervention*" OR "initiative*" OR "project*" OR "scheme*" OR "model*" OR "training") OR abstract("program*" OR "intervention*" OR "initiative*" OR "project*" OR "scheme*" OR "model*" OR "training"))) OR ((title("parenting") AND title("program*" OR "intervention" OR "initiative" OR "project" OR "model" OR "training" OR "class*" OR "education")) AND title("financ*" OR "microfinanc*" OR "debt" OR "money" OR "income" OR "economic" OR "cash" OR "budget*" OR "voucher" OR "expenditure" OR "cost" OR "goods")) OR ((title(("home visit" OR "home visitation" OR "home visiting" OR "home visitor" OR "home visits")) AND title(parenting OR parents OR parent OR mother* OR father* OR family OR families OR child*)) AND title("financ*" OR "microfinanc*" OR "debt" OR "money" OR "income" OR "economic" OR "cash" OR "budget*" OR "voucher" OR "expenditure" OR "cost" OR "goods") AND (title("program*" OR "intervention*" OR "initiative*" OR "project*" OR "scheme*" OR "model*" OR "training") OR abstract("program*" OR "intervention*" OR "initiative*" OR "project*" OR "scheme*" OR "model*" OR "training")))

Web of Science

**parenting or families or family** (Title) and **program* or intervention or initiative or project or model or training or class* or education** (Title) and **support* or help* or assist* or guidance or advice or counselling** (Title) and **financ* or money or debt or household or income or wellbeing or concrete or material or food** (Title)

Programme title search

Searched for title on EMBASE and Social Policy and Practice and PsycINFO.

- Added (parent or parenting or parents or mother* or father* or family or families).ti. if name of the programme was too generic.
- Added (Financ* or debt or welfare or money).ti,ab. if number returned was still 100+

**Online Resource 4: Programme registries searched**

| **Registry** | **Website** |
| --- | --- |
| Blueprints for Healthy Youth Development | <https://blueprintsprograms.org> |
| California Evidence-Based Clearinghouse for Child Welfare | <https://www.cebc4cw.org> |
| Clearinghouse for Military Family Readiness | <https://militaryfamilies.psu.edu> |
| Early Intervention Foundation Guidebook | <https://guidebook.eif.org.uk/> |
| Home Visiting Evidence of Effectiveness | <https://homvee.acf.hhs.gov> |
| Office of Juvenile Justice and Delinquency Prevention Model Programs Guide | <https://ojjdp.ojp.gov/model-programs-guide/> |
| Results First Clearinghouse | <https://evidence2impact.psu.edu/results-first-resources/clearing-house-database/> |
| Xchange | <https://www.euda.europa.eu/best-practice/xchange_en> |

**Online Resource 5: Programme registry searches**

| **Registry name** | **Filters (if applicable)** | **Searches (if applicable)** | **Number of results** | **Intervention identified by EB as potentially relevant** | **Agreed by GS and NA to include in stage iv search** |
| --- | --- | --- | --- | --- | --- |
| Blueprints for Healthy Youth Development | N/A | Financ | 8 | Nurse Family Partnership | Yes |
|  |  | Income | 45 | None relevant |  |
|  |  | Cash | 0 | N/A |  |
|  |  | Economic | 30 | None relevant |  |
|  |  | Budget | 0 | N/A |  |
|  |  | Concrete | 2 | None relevant |  |
| California Evidence-Based Clearinghouse for Child Welfare (CEBC) | N/A | Financ | 16 | Parents Under Pressure (PuP) | Yes |
|  |  |  |  | InsideOut Dad | Yes |
|  |  | Income | 18 | LIFE - Live In Family Enhancement | Yes |
|  |  | Cash | 0 | N/A |  |
|  |  | Economic | 22 | Building Healthy Children (BHC) | Yes |
|  |  |  |  | Boys Town In-Home Family Services (BT-IHFS) | Yes |
|  |  | Budget | 2 | None relevant |  |
|  |  | Concrete | 18 | Families First of Michigan | Yes |
|  |  |  |  | C.A.R.E.S. (Coordination, Advocacy, Resources, Education and Support) | Yes |
|  |  |  |  | Indiana Family Preservation Services | Yes |
| Clearinghouse for Military Family Readiness: Continuum of Evidence | Topic: Financial literacy | N/A | 51 | InsideOut Dad® | As above (duplicate) |
|  |  |  |  | Project Fatherhood | Yes |
| Early Intervention Foundation Guidebook | Program specifics: Parent training | Financ | 0 | None relevant |  |
|  |  | Income | 9 | Raising Healthy Children | Yes |
|  |  | Cash | 1 | None relevant |  |
|  |  | Economic | 13 | None relevant |  |
|  |  | Budget | 6 | None relevant |  |
|  |  | Concrete | 2 | None relevant |  |
| Home Visiting Evidence of Effectiveness (HomVEE) | Family economic self-sufficiency | N/A | 6 | None relevant |  |
| Office of Juvenile Justice and Delinquency Prevention Model Programs Guide | N/A | Financ | 0 | N/A |  |
|  |  | Income | 12 | None relevant |  |
|  |  | Cash | 0 | N/A |  |
|  | Filter only used for 'economic' search. 'adult 18+' took from 63 to 33 results | Economic | 33 | None relevant |  |
|  | N/A | Budget | 0 | N/A |  |
|  |  | Concrete | 0 | N/A |  |
| Results First Clearinghouse | Child & family wellbeing OR Employment & job training | Financ | 38 | Support Groups for Grandparent Caregivers of Children with Developmental Disabilities and Delays | Yes |
|  |  | Income | 83 | Early Head Start | Yes |
|  |  |  |  | Nurse Family Partnership | As above (duplicate) |
|  |  | Cash | 2 | None relevant |  |
|  |  | Economic | 31 | None relevant |  |
|  |  | Budget | 2 | None relevant |  |
|  |  | Concrete | 7 | Friends of the Children (FOTC) | Yes |
| Xchange | N/A | Financ | 0 | N/A |  |
|  |  | Income | 1 | Nurse Family Partnership | As above (duplicate) |
|  |  | Cash | 0 | N/A |  |
|  |  | Economic | 0 | N/A |  |
|  |  | Budget | 0 | N/A |  |
|  |  | Concrete | 0 | N/A |  |

**Online Resource 6: Data items**

| **Main category** | **Sub-category** | **Dropdown options (if applicable)** |
| --- | --- | --- |
| Included studies | First author (family name) |  |
|  | Country of institution |  |
|  | Date published |  |
|  | Full reference |  |
|  | Extracted by (initials) – Person 1 |  |
|  | Extracted by (initials) – Person 2 |  |
|  | Extracted date – Person 1 |  |
|  | Extracted date – Person 2 |  |
|  | Checked by (initials) |  |
|  | Checked date |  |
| Study characteristics | Paper title |  |
|  | Country of intervention |  |
|  | Dates that study ran (or duration, if not reported) |  |
| Evaluation method | Study design | Pre post |
|  |  | RCT |
|  |  | Quasi-experimental |
|  |  | Qualitative |
|  |  | Mixed methods |
|  |  | Other |
|  | Type of evaluation | Efficacy/effectiveness |
|  |  | Feasibility |
|  |  | Implementation |
|  |  | Effectiveness-implementation hybrid |
|  |  | Other |
|  | Participant number (adults and CYP) |  |
|  | Control / service as usual participants (N) |  |
|  | Intervention participants (N) |  |
|  | Sample (including age, SES, ethnicity, gender) |  |
| Measures | Data collection methods | Interviews |
|  |  | Focus groups |
|  |  | Survey (self-report) |
|  |  | Survey (third party-reported) |
|  |  | Secondary data (e.g. examination of routine data) |
|  |  | Observation (including ethnography) |
|  |  | Other |
|  | Financial outcomes | Income |
|  |  | Debt |
|  |  | Financial stress |
|  |  | Financial control |
|  |  | Material living standards |
|  |  | Employment |
|  |  | Other |
|  |  | None |
|  | Parent health and wellbeing outcomes | Mental health |
|  |  | Physical health |
|  |  | Alcohol / drug use |
|  |  | Relationship with partner |
|  |  | Other |
|  |  | None |
|  | Parenting outcomes | Knowledge |
|  |  | Skills |
|  |  | Stress (parenting related) |
|  |  | Relationship with child |
|  |  | Safety and basic care |
|  |  | Other |
|  |  | None |
|  | Child outcomes | Physical health |
|  |  | Physical development |
|  |  | Psychological/ emotional development |
|  |  | Mental health |
|  |  | Intellectual/ cognitive development |
|  |  | Educational |
|  |  | Behavioural |
|  |  | Social |
|  |  | Other |
|  |  | None |
|  | Implementation outcomes | Acceptability |
|  |  | Adoption |
|  |  | Appropriateness |
|  |  | Cost |
|  |  | Fidelity |
|  |  | Feasibility |
|  |  | Penetration |
|  |  | Sustainability |
|  |  | Other |
|  |  | None |
|  | Name of financial outcome used |  |
|  | Analysis methods/stats |  |
| Intervention | Programme name |  |
|  | Aims of intervention (e.g., to increase income) |  |
|  | Target group (e.g. age of adults/children, income, previous incarceration, receiving of a government grant, number of children or employment status etc. OR universal); inclusion and exclusion criteria |  |
|  | Delivery setting (school, home, community etc.) | Home |
|  |  | School / early years setting |
|  |  | Community (not school / early years) |
|  |  | Primary care |
|  |  | Other |
|  | Frequency/ duration of sessions |  |
|  | Provider (organisation e.g., NHS, third sector agency) |  |
|  | Provider (professions e.g., midwife, family support worker, paraprofessional) |  |
|  | Parenting support topic / content | Parent-child relationship |
|  |  | Parent-partner Relationship |
|  |  | Family functioning |
|  |  | Safety and basic care |
|  |  | Managing conflict |
|  |  | Managing behaviour |
|  |  | Other |
|  | Financial wellbeing support topic / content | Provision of money (e.g., cash, vouchers, loans) |
|  |  | Provision of goods (e.g., furniture, white goods, food, clothes) |
|  |  | Support to reduce debt |
|  |  | Support to secure benefit entitlements |
|  |  | Support with money management (e.g., budgeting financial literacy) |
|  |  | Support to boost income through work (e.g., loans, training, education) |
|  |  | Other |
|  | Other programme components | In-person group |
|  | Mode of delivery | In-person individual |
|  |  | Online group |
|  |  | Online individual |
|  |  | Online resource (no facilitator / participants e.g., recorded videos) |
|  |  | Other |
|  | Brief description of logic model / theory of change (in particular FWbS x PS) |  |
| Notes |  |  |

**Online Resource 7: Narrative summary of outcome results for each study**

| **Study / Design** | **Financial outcomes** | **Parent health/well-being outcomes** | **Parenting outcomes** | **Child health/development outcomes** |
| --- | --- | --- | --- | --- |
| Benzies et al. (2009)  Pre-post | Statistically significant increase in daily life management skills (which includes budgeting). | Statistically significant increase in parent self-esteem. | Statistically significant reduction in some aspects of parenting-related stress, notably parental distress and defensive responding. | N/A |
| Cluver et al. (2018)  RCT | Significant intervention effects of reduced past-month household economic hardship (caregiver and adolescent reports) and improved past-month family financial management, including reduced past-month borrowing and increased savings (caregiver report, adolescent report not measured). | Caregivers reported significant intervention effects of reduced depression and alcohol/substance abuse. | Caregivers reported significant intervention effects of reduced parenting stress. On both caregiver and adolescent reports, those receiving the parenting intervention had reduced physical and emotional abuse (at least in the short term) and improved involved parenting and parental supervision. Significant intervention effects of reduced corporal punishment and less attitudes condoning corporal punishment (both caregiver report only). No intervention effects on neglect or inconsistent discipline (caregiver and adolescent reports). | Significant intervention effect of reduced adolescent self-reported substance use but there were no intervention effects on adolescent- and caregiver-reported externalising behaviour or adolescent-reported depression/suicidality. |
| Dawe et al. (2003)  Pre-post | N/A | Positive changes in parents’ substance abuse and risk-taking. Three of the eight parents followed-up showed clinically significant improvements on an HIV risk-taking behaviour scale and a clinically significant decrease in methadone dose. Six parents showed clinically significant improvements on a measure of alcohol use. | Significant decreases for whole sample on all three subscales of a parenting stress measure (parental distress [including relationship with partner], perception of child as difficult to manage, satisfaction with parent-child interactions), with almost all parents showing clinically significant improvement. Substantial decrease in the overall score on a measure of physical abuse potential. | Positive impact on child behaviour, with parents reporting fewer behavioural problems on a self-report behaviour checklist (behaviours included *inter alia* temper outbursts, unpredictable behaviour and mood changes). Clinically significant parent-reported decrease in child behaviour problems, with most parents showing a clinically significant improvement. |
| Katz et al. (2011)  RCT | N/A | N/A | Intervention participants improved their home environments (conducive to good child development). No significant between-group differences on measures of parenting stress, parent knowledge of infant development, or child-rearing attitudes associated with child maltreatment. | No significant between-group differences on measures of infant development (mental, psychomotor, behaviour). |
| Lachman et al. (2020)  Cluster RCT | Borderline increases in basic child necessities reported by children in the combined intervention but no significant effects in any intervention arms on household hunger, child food consumption, agricultural production, or family budgeting. | No significant effects in intervention arms on parent depression or intimate partner violence. No analysis of parent alcohol use due to extremely low reports of alcohol use at baseline. | No significant effect in intervention arms on parenting stress. Significant reductions in parent-reported overall child maltreatment, also child-reported overall maltreatment (latter combined intervention only). Results for discrete types of abuse by source were mixed. Less parent endorsement of corporal punishment in the combined intervention. | Significant reductions in parent-reported child behaviour problems in all intervention arms but no effects for child depression, positive child behaviour, child sexual behaviour or early childhood development outcomes. Borderline effects in the combined arm for measurements of mid-upper arm circumference for children aged 0-3 years. No analysis of child alcohol use due to extremely low reports of alcohol use at baseline. |
| Mathias et al. (2022)  Mixed methods process and feasibility evaluation | N/A | N/A | N/A | N/A |
| Mebrahtu et al. (2019)  Cluster RCT | No effect on household food security. | No effect on maternal physical health measured in terms of HIV viral load or on maternal mental health measured in terms of postnatal depression or risk for common mental disorders. | Strong evidence that the programme had a positive impact on one aspect of parenting stress, namely parental distress. | Mixed impact on child nutritional outcomes; no impact on weight or height for age or body mass index, but strong evidence of a positive impact on mid-upper arm circumference. No impact on global child development (early learning composite and visual, motor and language subscales). Numbers too small to detect any difference between trial arms in child HIV prevalence or viral suppression. |
| Miller et al. (2014)  Effectiveness/ implementation hybrid | N/A | N/A | Statistically significant reduction in endorsement of corporal punishment attitudes. No statistically significant change on other subscales of a measure of parenting attitudes associated with risk for child maltreatment (e.g., inappropriate developmental expectations, lack of empathy). | N/A |
| Paradis et al. (2013)^1^  RCT | Educational and employment gains for parents (no data presented). | Of the parents referred to therapy for maternal depression, 60% engaged with therapy and reduced depressive symptoms. Of parents referred to therapy for maternal-child attachment, 79% connected with services and achieved treatment goals. | Statistically significant higher completion rate for well-child visits. Higher rate avoiding Child Protection Service reports (not statistically significant). | No data or results reported |
| Sadler et al. (2018)  Qualitative | N/A | N/A | N/A | N/A |
| Wimer et al. (2021)^2^  RCT | Statistically significant moderate to large effects for both toys and goods and number of books in the home. No statistically significant effect on financial worries. | No significant effects on maternal depressive symptoms or perceived stress (latter approached statistical significance and favoured intervention group mothers). | Strongest significant effect for parental sense of competence. Chaos in the home and engagement in cognitively stimulating activities approached statistical significance and favoured the intervention group. Statistically significant effects for aggravation in parenting, a measure of stress experienced by parents associated with caring for children. | N/A |

^1^ Preliminary analyses as evaluation was ongoing (not all participants had completed programme).

^2^ Analysis of proximal outcomes after 12 months (programme lasts 3 years).

1. Page numbers refer to manuscript in original submission.
